# Integrated single-cell and bulk RNA sequencing analysis identifies a prognostic signature related to ferroptosis dependence in colorectal cancer

**DOI:** 10.1038/s41598-023-39412-y

**Published:** 2023-08-04

**Authors:** Xiaochen Xu, Xinwen Zhang, Qiumei Lin, Yuling Qin, Yihao Liu, Weizhong Tang

**Affiliations:** 1 Department of Gastrointestinal Surgery, Guangxi Medical University Cancer Hospital, Nanning, 530021 Guangxi Zhuang Autonomous Region China; 2 Department of Clinical Laboratory, Guangxi Medical University Cancer Hospital, Nanning, 530021 Guangxi Zhuang Autonomous Region China; 3 Department of Gastrointestinal Surgery, Affiliated Hospital of Guilin Medical University, Guilin, 541001 Guangxi Zhuang Autonomous Region China

**Keywords:** Gastrointestinal cancer, Cell death

## Abstract

Ferroptosis is an iron-dependent form of cell death induced by lipid oxidation with an essential role in diseases, including cancer. Since prognostic value of ferroptosis-dependent related genes (FDRGs) in colorectal cancer (CRC) remains unclear, we explored the significance of FDRGs in CRC through comprehensive single-cell analysis. We downloaded the GSE161277 dataset for single-cell analyses and calculated the ferroptosis-dependent gene score (FerrScore) for each cell type. According to each cell type-specific median FerrScore, we categorized the cells into low- and high-ferroptosis groups. By analyzing differentially-expressed genes across the two groups, we identified FDRGs. We further screened these prognosis-related genes used to develop a prognostic signature and calculated its correlation with immune infiltration. We also compared immune checkpoint gene efficacy among different risk groups, and qRT-PCR was performed in colorectal normal and cancer cell lines to explore whether the signature genes could be used as clinical prognostic indicators. In total, 523 FDRGs were identified. A prognostic signature including five signature genes was constructed, and patients were divided into two risk groups. The high-risk group had poor survival rates and displayed high levels of immune infiltration. Our newly developed ferroptosis-based prognostic signature possessed a high predictive ability for CRC.

## Introduction

Of the most common cancer types, colorectal cancer (CRC) accounts for 9.4% of all cancer deaths worldwide^1^. Currently, surgery and chemotherapy have significantly improved the survival rates of CRC patients^2^. However, owing to a lack of efficient clinical treatment and prognostic biomarkers, the overall prognosis of CRC is poor. Moreover, because of tumor heterogeneity, the clinical and histopathological features of tumors cannot currently be used to accurately predict the course of CRC. Therefore, it is critical to identify new prognostic factors and treatment targets for CRC.

Over the past few years, tumor heterogeneity has been shown to be a significant challenge in the treatment and prognosis of cancer. Recently, single-cell RNA sequencing (scRNA-seq) has attracted considerable attention. It allows for the genome-wide analysis of individual cells and makes it possible to understand cellular heterogeneity^3,4^. Li et al.^5^ compared the intra-tumor cell heterogeneity between carcinoma and normal tissues in CRC using scRNA-seq. Poonpanichakul et al.^6^ used a droplet-based scRNA-seq method to profile intra-tumor cell heterogeneity in CRC ascites. However, few studies have been conducted on the cellular heterogeneity of CRC during its evolution from adenoma to carcinoma. In this study, we analyzed the cellular heterogeneity of adenomas and carcinomas by single-cell analysis, and used this information to develop an effective treatment strategy for CRC.

Ferroptosis, an iron-dependent cell death process, is characterized by lipid peroxidation. It is morphologically and mechanistically distinct from the other types of cell death^7^. Increasing evidence suggests that ferroptosis plays a role in various cancers^8^. Lu et al.^9^ found that downregulation of KLF2 inhibits ferroptosis by reducing the transcriptional repression of GPX4 and promoting the invasive activity of renal cell carcinoma. Moreover, because iron metabolism and homeostasis are associated with tumor immunity, they also play a significant role in immunity^10^. Wang et al.^11^ demonstrated that the activation of CD8^+^ T cells could increase ferroptosis and the efficacy of immunotherapy. However, the mechanisms underlying ferroptosis in CRC, and the role that ferroptosis-dependent related genes (FDRGs) of CRC remain unclear. Therefore, it is necessary to understand the pathophysiology and underlying mechanisms of FDRGs in CRC.

In this study, we calculated ferroptosis-dependent gene scores (FerrScores) for different CRC cell types and used them to define FDRGs. We also constructed a prognostic signature for CRC. Our findings may provide a novel therapeutic strategy for CRC.

## Methods

### Data collection

To compare the cell heterogeneity in colorectal adenoma and carcinoma tissues, we downloaded the GSE161277 CRC sequence library from the Gene Expression Omnibus database (GEO) (http://www.ncbi.nlm.nih.gov/geo/) and selected four colorectal adenoma and four colorectal carcinoma samples (GSM4904234, GSM4904235, GSM4904236, GSM4904238, GSM4904239, GSM4904242, GSM4904243, and GSM4904245) for single-cell analysis^12^. We also downloaded the RNA-Seq data and relevant clinical information on CRC from the official website of The Cancer Genome Atlas (TCGA) (https://portal.gdc.cancer.gov/). Furthermore, we obtained the validation dataset GSE17538 from the GEO database. A list of ferroptosis-dependent genes was compiled using the FerrDb website (http://www.zhounan.org/ferrdb/current/).

### Processing of sc-RNAseq data

We used the “Seurat” R package (version 4.1.1) and integrated downstream analysis of single-cell transcriptome profiles. The data were quality controlled. Cells with fewer than 300 features and genes expressed in fewer than three cells were excluded. In addition, the proportion of mitochondria was limited to less than 20%. We then normalized the data using the LogNormalization method. We also screened the 2000 highly variable genes with the “FindVariableFeatures” function. Uniform manifold approximation and projection (UMAP) was used for data visualization in 2 dimensions^13^. Subsequently, the “FindNeighbors” function was used for cell clustering analysis. Furthermore, we used the FindAllMarkers function to define genes in each cluster. Finally, the “SingleR” package was employed for cell-type annotation. We also validated the annotation via cell markers from a previous study^14^.

We loaded 259 ferroptosis-dependent genes and calculated FerrSore in each cell using the AddModuleScore function. We also compared the FerrScore between adenoma and carcinoma samples. According to each cell type-specific median FerrScore, we divided these cells into low-ferroptosis and high-ferroptosis cell groups. We also used the FindMarker function to screen out the differentially-expressed genes (DEGs) of the two groups (adjusted *p* < 0.05, |log _twofold_ change) |> 0.5). We defined these genes as FDRGs.

We explored the cell ratios of the adenoma and carcinoma samples of the low and high-ferroptosis cell groups. We also performed gene set variation analysis (GSVA) to analyze the enrichments of different cell types in adenoma and carcinoma samples based on the “GSVA” R package^15^.

### Processing of TCGA data

We used the Perl programming language to extract RNA-Seq data from the database. The expression data of the FDRGs were then extracted based on the TCGA data. Patients with short survival times (less than 30 days) or those who died were excluded from the clinical data.

### FDRG prognostic signature construction and validation

Patients were randomly divided into training and test cohorts in a 1:1 ratio (Table 1). For the training cohort, we performed univariate Cox regression analysis to identify genes associated with prognosis. In order to minimize the potential of overfitting, we employed Least Absolute Shrinkage and Selection Operator (LASSO) Cox proportional hazards regression to evaluate prognostic genes^16^. Subsequently, a stepwise multivariate Cox regression analysis was conducted using the genes identified through LASSO Cox regression. This analysis aimed to ascertain the prognostic significance of specific gene signatures. Finally, a risk model was constructed by combining the mRNA expression of the genes with their respective risk coefficients in a linear fashion.

**Table 1:** Clinical characteristics of CRC patients involved in the study.

Covariates	Type	Total	Test	Train	*p*-value
Age	< = 65	196	97	99	0.9234
	> 65	242	122	120	
Gender	Female	197	106	91	0.1787
	Male	241	113	128	
Stage	Stage I	78	38	40	0.8611
	Stage II	160	78	82	
	Stage III	119	61	58	
	Stage IV	66	36	30	
	unknow	15	6	9	
T	T1	13	8	5	0.3971
	T2	81	38	43	
	T3	299	146	153	
	T4	45	27	18	
M	M0	329	162	167	0.4415
	M1	65	36	29	
	unknow	44	21	23	
N	N0	254	124	130	0.6727
	N1	108	54	54	
	N2	75	41	34	
	unknow	1	0	1	

The median risk score was used to categorize all patients into high- and low-risk categories. Risk scores were calculated by multiplying gene expression levels by the coefficients of the signature genes. Kaplan–Meier survival curves were plotted using the “survival” R package. A receiver operating characteristic (ROC) curve for the signature was calculated and displayed using the R package “time-ROC”. In addition, we performed univariate and multivariate Cox proportional hazards analyses in the TCGA cohort to determine the predictive value of the riskscore when combined with clinical variables such as age, gender, and stage. We further assessed the association between the riskscore and patient survival outcomes. we also examined the differential expression levels of genes within the signature between the different risk groups. Finally, we conducted nomogram with Risk scores and clinicopathological features to determine the efficacy of Overall Survival (OS) rates at 1-, 2-, and 3-year in CRC patients.

### Analysis of functional enrichment

To determine the biological functions of the signature, we analyzed the Gene Ontology (GO) and Kyoto Encyclopedia of Genes and Genomes (KEGG) data using the ClusterProfiler R package^17^. Enrichment was defined as a *p*-value of < 0.05.

### Correlation between immunity infiltration and signatures

Single-sample gene set enrichment analysis (ssGSEA) was used to detect the level of immune cell infiltration in correlation with the signature. Immune cell infiltration scores for the low- and high-risk groups were calculated using the “GSVA” R package. Moreover, we compared the immune function between different risk groups.

Based on the TCGA dataset, we calculated immune scores, stromal scores, and ESTIMATE scores using the “estimated” R package^18^. Moreover, we compared the risks in the different groups. Finally, the expression of checkpoint genes^19–22^ in the two risk groups was compared.

### CRC cell line culture and quantitative real-time polymerase chain reaction (qRT‑PCR)

Human normal intestinal epithelial cells (NCHM460) were obtained from IMMOCELL (Xiamen,China), and human colon cancer cell lines (Caco2, HCT15, HCT116, HT29, Lovo, SW480, SW620) were obtained from iCell (Shanghai, China). All these cells were cultured in media containing 10% fetal bovine serum (FBS) and 1% penicillin/streptomycin (P/S) at 37 °C in a humidified atmosphere of 5% CO2. The culture media used were DMEM, MEM, 1640, McCOY’s 5A, McCOY’s 5A, F12K, L15 and L15, which were purchased from Gibco BRL in the USA.

Total cellular and tissue RNA was extracted from tissues or cell lines using Total RNA Extraction Reagent (DP451, Tiangen) according to standard protocols. The obtained RNA was then used for cDNA synthesis using cDNA Synthesis Kit (MR05401S, Monad). Gene expression was quantified using SYBR Green Master Mix (MQ10301S, Monad) on a Roche LightCycler 480 and expression levels were calculated using the 2^−ΔΔCT^ method.

GAPDH was used as an internal reference for normalization. All primers used for qRT-PCR were synthesized by Wuhan Jinkairui Bioengineering Co Ltd (Wuhan, China). The sequences of the primers used are listed in Table 2.

**Table 2:** The sequences of the qRT-PCR primer used in this study.

Gene	Forward primer	Reverse primer
GAPDH	GAGTCAACGGATTTGGTCGT	GACAAGCTTCCCGTTCTCAG
TIMP1	AGTTTTGTGGCTCCCTGGAA	TCCGTCCACAAGCAATGAGT
MANF	ATATCGGGGCCACAGATGATG	AACTCGGAGCTTCTTCAGGTC
HSPA1A	CCTGTTTGAGGGCATCGACT	TCGTGAATCTGGGCCTTGTC
RPS17	GTTCGCACCAAAACCGTGAA	GCTTGTTCGTGTGGAAGTCG
PTMA	CCTGCTAACGGGAATGCTG	CGTCGGTCTTCTGCTTCTTG

### Statistical methods

We analyzed the data using R software (version 4.1.3) and compared the differences between the groups the Wilcoxon test. Spearman’s correlation coefficient was also calculated; *p* values of < 0.05 were considered statistically significant.

## Results

### Single-cell clustering and acquisition of FDRGs

**Figure 1:**
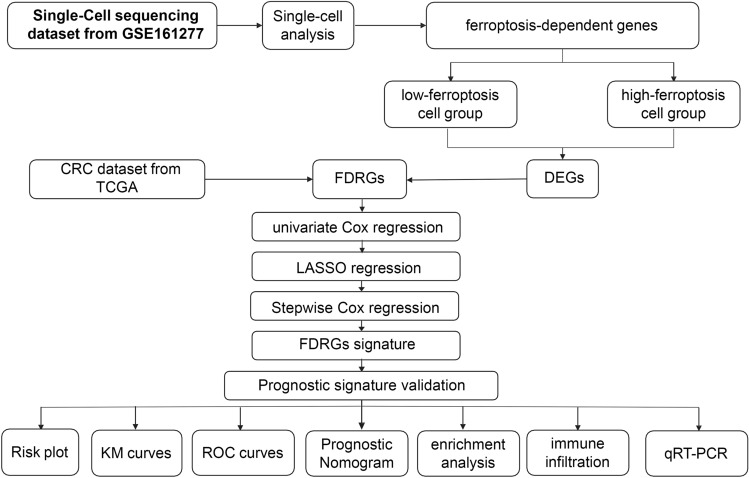
Data collection and analysis in this study.

**Figure 2:**
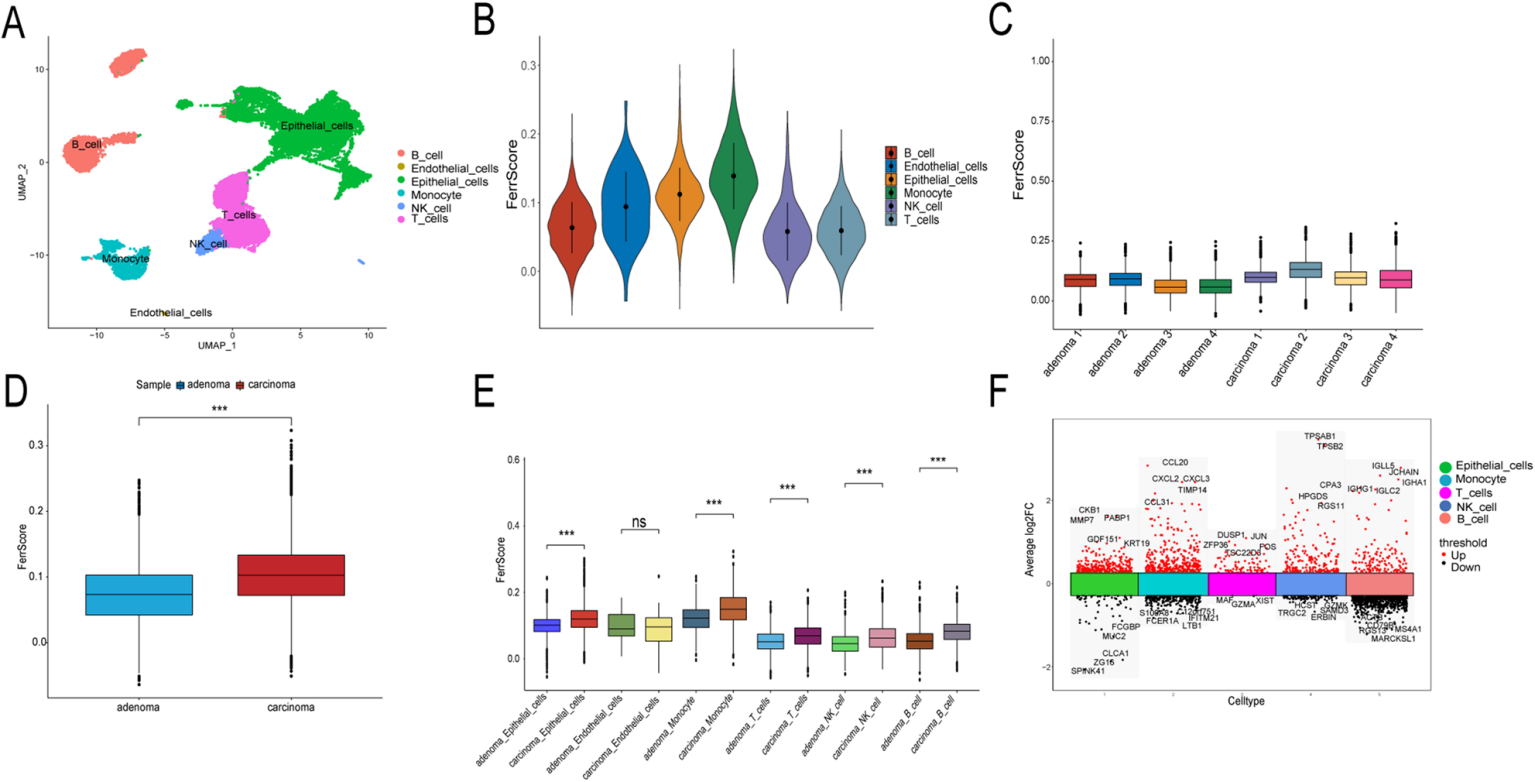
Single-cell RNA sequencing analysis of 24,732 cells from four colorectal adenomas and four colorectal carcinoma tissues. (**A**) Cells were clustered into six types by a UMP dimensionality reduction algorithm, and each color represented the annotated phenotype of each cluster. (**B**) The ferroptosis-dependent genes scores (FerrScores) of the six cell types. (**C**) Comparison of FerrScores for each sample. (**D**) Comparison of FerrScores between adenoma and carcinoma groups. (**E**) Comparison of FerrScores for different cell types in adenoma and carcinoma samples. (**F**) Top five differential expressed genes (DEGs) with log2FC values. *p < 0.05, **p < 0.01, ***p < 0.001, ****p < 0.0001.

The overall research design is illustrated in Fig. 1. A total of 24,732 high-quality cells were obtained from the eight samples after filtering and quality control (Supplementary Fig. 1A). There was no significant correlation between the sequencing depth (total number of UniqueMolecular Identifiers, nCount) and mitochondrial percentage (Pearson correlation coefficient r = 0.18); however, nCount was positively correlated with the number of detected genes (nFeature) (r = 0.92) (Supplementary Fig. 1B). Subsequently, the top ten highly variable genes out of 2000 were marked in red (Supplementary Fig. 1C). Twenty-three cell clusters were identified and visualized in two dimensions (Supplementary Fig. 2A). The cell identity of each cluster was annotated to six cell types. These cell types were characterized as epithelial cells, monocytes, endothelial cells, B-cells, NK-cells, and T-cells (Fig. 2A).

Bubble plots were used to show the average expression levels of representative marker genes for different cell types in the integrated human CRC data^23^ (Supplementary Fig. 2B).

The FerrScores of the different cell types are shown in Fig. 2B. Figure 2C shows the FerrScore of each sample. We also compared the FerrScore among adenoma and carcinoma samples. The differences in FerrScore between adenoma and carcinoma samples were significant. (Fig. 2D, ***p < 0.001). Figure 2E shows a comparison of the FerrScore for different cell types in adenoma and carcinoma samples. Among these cell types, there were significant differences between adenoma and carcinoma samples, except for endothelial cells (***p < 0.001).

For each cell type, we divided these cells into a low-ferroptosis and a high-ferroptosis cell group based on the cell type-specific median FerrScore. Moreover, 523 DEGs were screened in the two groups. We also identified the top five genes with log2FC values (Fig. 2F).

### Cell ratios of the samples and functional enrichment by GSVA

**Figure 3:**
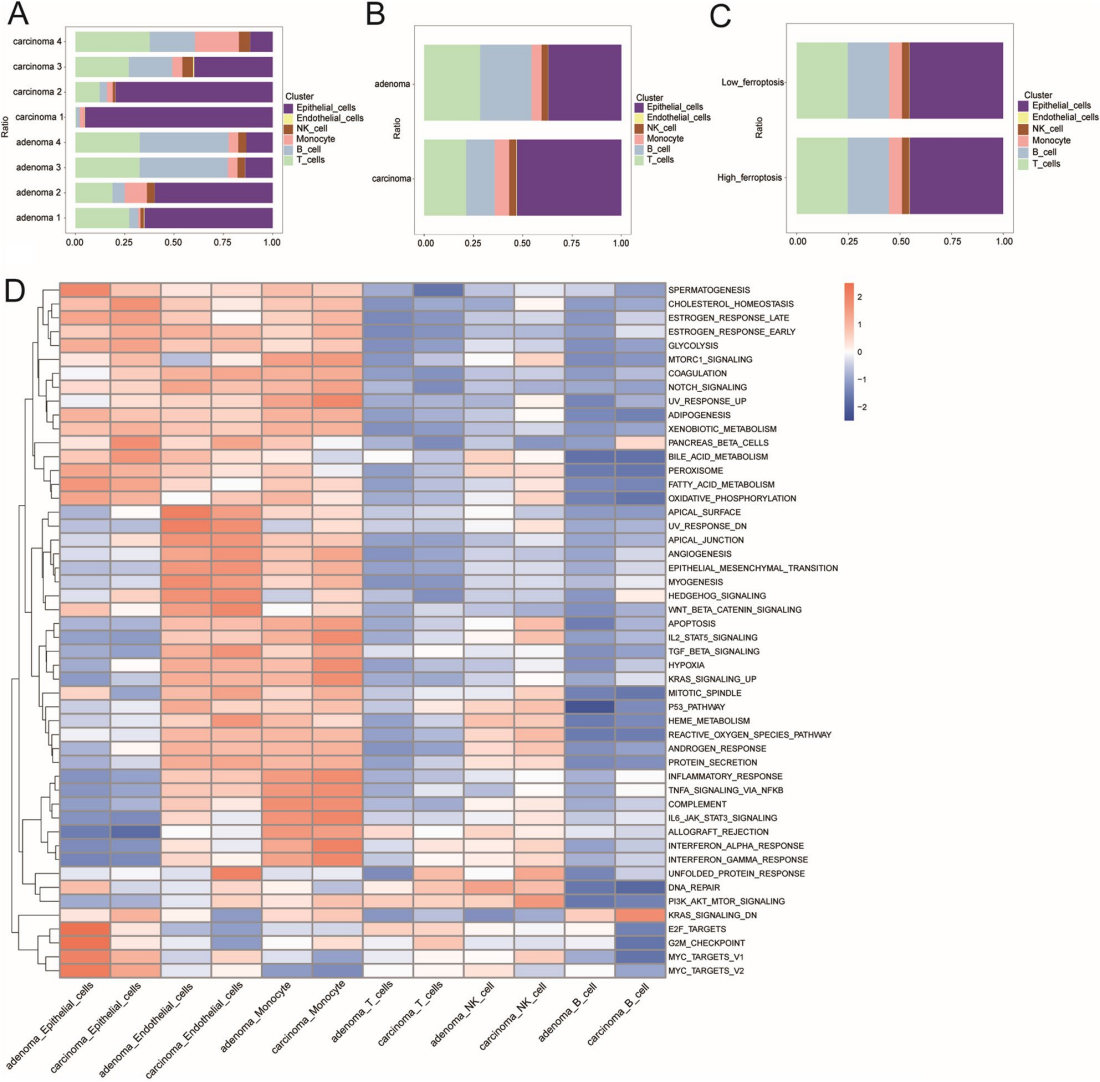
Cell ratios of different samples and GSVA analysis. (**A**) Cell ratios of each sample. (**B**) Cell ratios of different cell types in adenoma and carcinoma samples. (**C**) Cell ratios of the high-ferroptosis and low-ferroptosis cell groups. (**D**) Results of GSVA of different cell types in adenoma and carcinoma.

The cell ratios for each sample are shown in Fig. 3A. We also show the ratios of the different cell types in terms of adenomas vs. carcinomas, in Fig. 3B. The cell ratios of the low-ferroptosis and high-ferroptosis cell groups are displayed in Fig. 3C. Figure 3D shows the results of gene set enrichment in the different cell types between adenoma and carcinoma samples.

### Construction of five-gene prognostic signature

**Figure 4:**
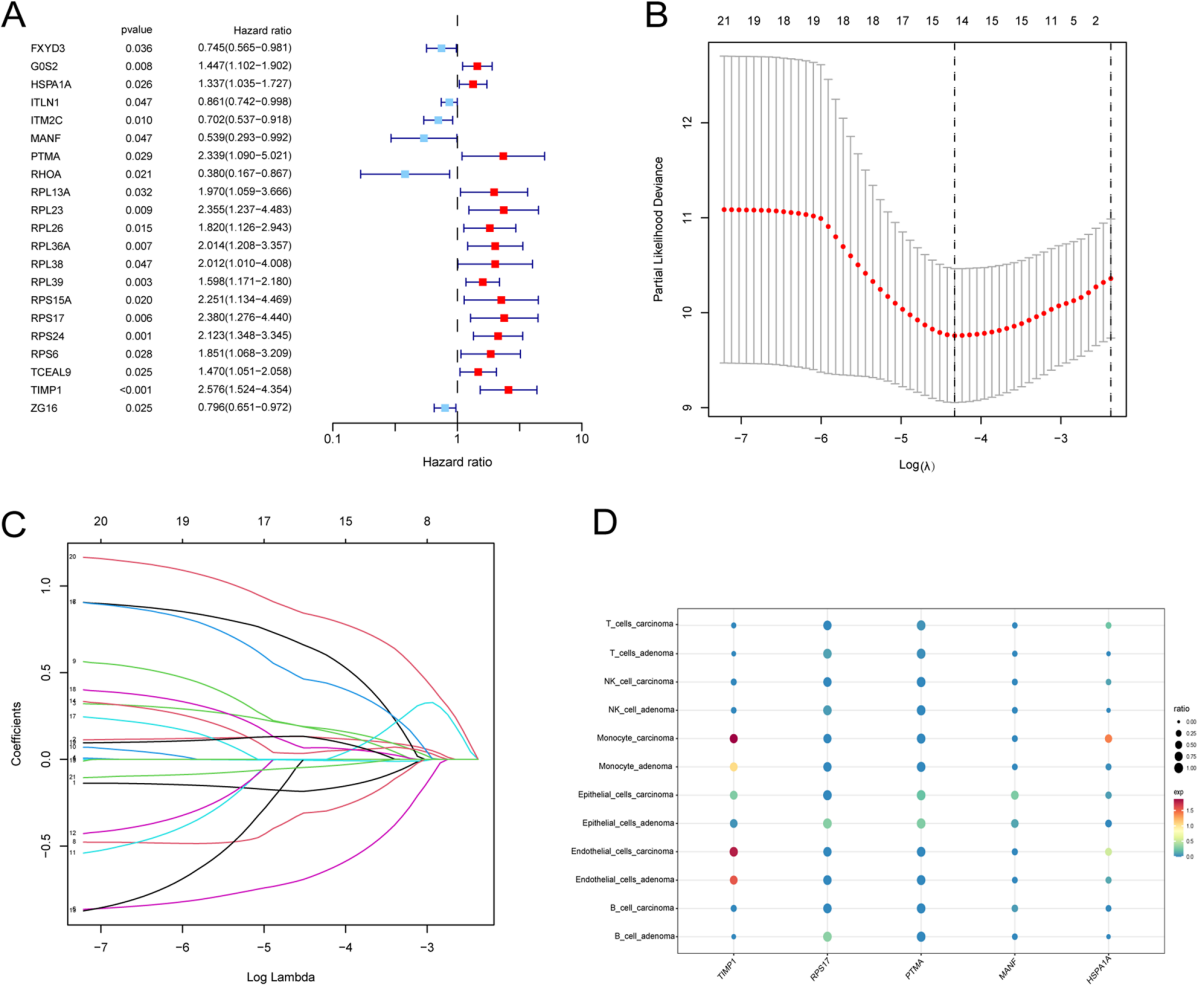
Construction of a prognostic signature. (**A**) Univariate Cox analysis of the TCGA cohort. (**B**–**C**) Using least absolute shrinkage and selection operator (LASSO) regression, a signature was constructed based on the optimum λ. (**D**) Bubble plot visualizing the expression of signature genes in cell types between adenoma and carcinoma samples.

We developed a prognostic signature based on FDRGs. Using univariate Cox regression analysis, we identified 21 genes associated with OS in patients with CRC (Fig. 4A). Then, LASSO regression analysis was performed on the 21 previously screened genes and obtained 14 genes by the optimal λ (Fig. 4B, C). Finally, we constructed a 5-gene (HSPA1A, MANF, PTMA, RPS17, and TIMP1) signature based on multifactorial stepwise Cox regression analysis. According to the formula, the riskscore = HSPA1A * 0.274668948995861 + MANF * (− 1.21739069354923) + PTMA * 1.07281342991973 + RPS17 * 0.820155083563555 + TIMP1 * 1.17800493596346 (Table 3). Figure 4D shows the expression of signature genes in the cell types between adenoma and carcinoma samples.

**Table 3:** The genes involved in the signature and their coefficients.

No	Gene name	Coef
1	T1MP1	1.17800493596346
2	PTMA	1.07281342991973
3	RPS17	0.820155083563555
4	HSPA1A	0.274668948995861
5	MANF	− 1.21739069354923

### Evaluation and validation of prognostic signature

**Figure 5:**
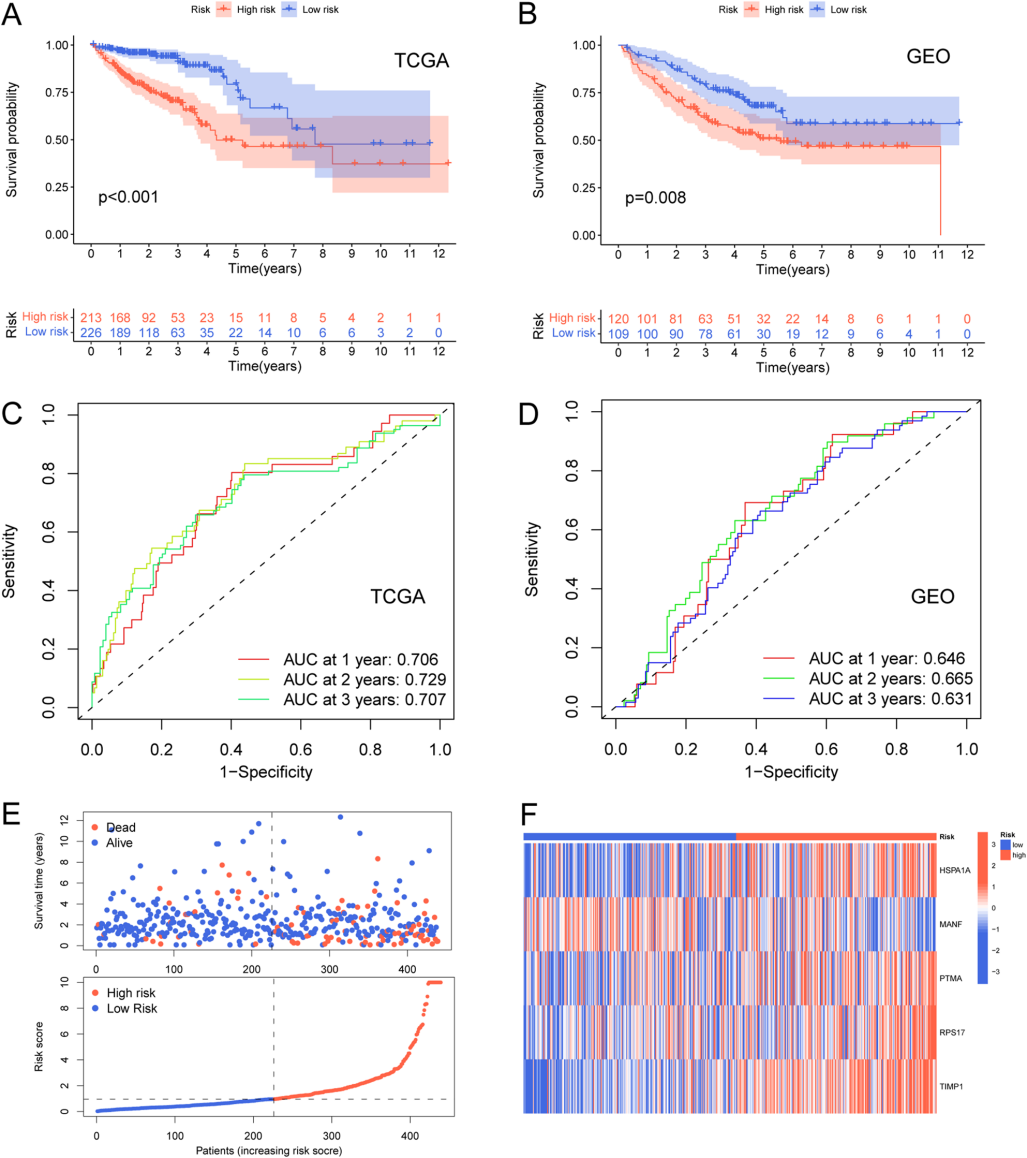
Validation of the prognostic signature. (**A**–**B**) Overall Survival (OS) for TCGA cohort and GEO cohort. (**C**–**D**) ROC curves of signatures in the TCGA cohort and GEO cohort. (**E**–**F**) Correlation of risk-scores and survival statuses of CRC patients.

As we validated the prognostic power of the signature, The Kaplan–Meier curves demonstrated that patients in the low-risk group had a significantly better overall survival (OS) compared to those in the high-risk group in both the TCGA and GEO cohorts (Fig. 5A–B). In the TCGA cohort, the area under the receiver operating characteristic (ROC) curves (AUCs) for 1-, 2-, and 3-year overall survival (OS) were 0.706, 0.729, and 0.707 respectively, as shown in Fig. 5C. The corresponding AUCs for the 1-, 2-, and 3-year OS in the GEO cohort were 0.646, 0.665, and 0.631 respectively, as presented in Fig. 5D. To further assess the predictive power of the risk traits, we conducted a comprehensive analysis that involved comparing the risk score distribution, survival time and status, and expression of model genes between the high-risk and low-risk groups Fig. 5E–F. After conducting both univariate and multivariate Cox proportional hazards analyses, we found that age and the riskscore were identified as independent prognostic factors, according to the results presented in Supplementary Fig. 3.

**Figure 6:**
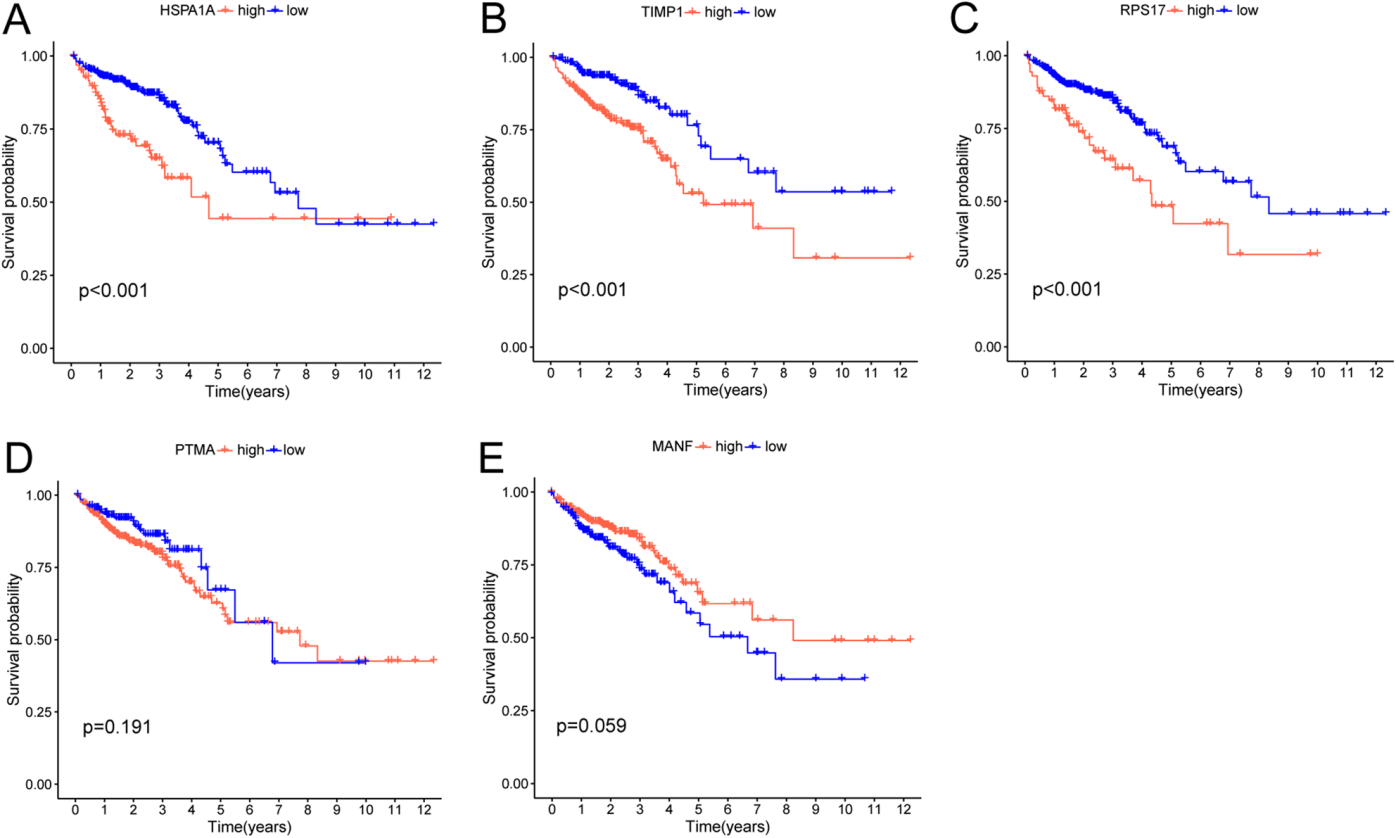
OS differences of different expression levels of genes in the signature (**A**–**E**). Lower expression of these genes displayed better OS, including HSPA1A, TIMP1, and RPS17, while PTMA and MANF's were not significantly associated with OS.

To investigate whether the signature genes have a prognostic significance, we divided them into high and low expression groups based on the optimal cutoff value. Lower expression of these genes displayed better OS, including HSPA1A, TIMP1, and RPS17, while PTMA and MANF's were not significantly associated with OS (Fig. 6A–E).

### Construction and Validation of nomogram

**Figure 7:**
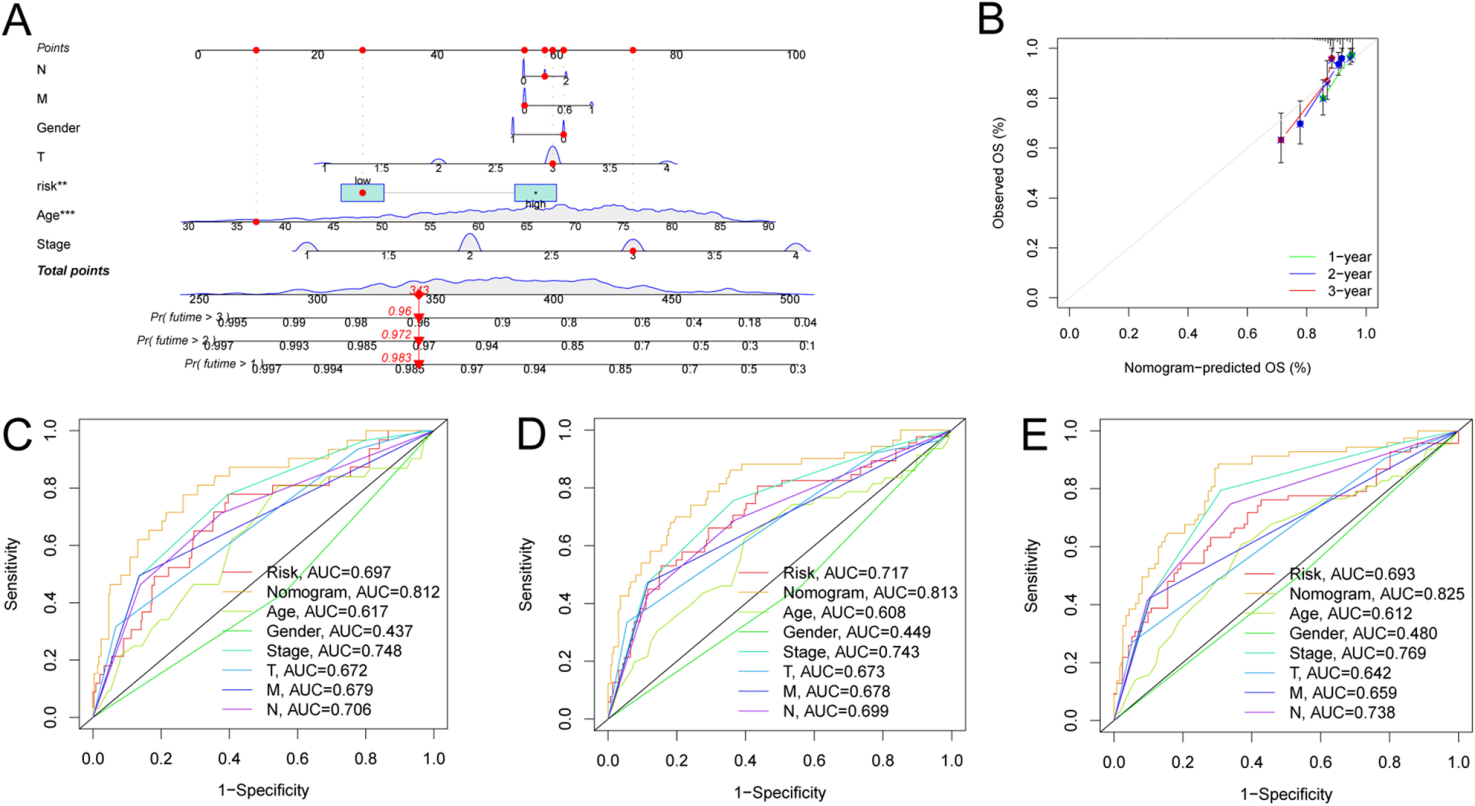
Nomogram construction. (**A**) A nomogram was constructed to facilitate prognosis prediction. Age, TNM, stage and risk-score variables were factored in the nomogram, and the total points indicated the survival probabilities of patients. (**B**) The calibration curve indicated that the predicted OSs by the nomogram were relatively consistent with the actual observed OSs. (**C**–**E**) The AUC values for diverse clinical factors, risk scores, and nomogram scores were determined via ROC curves at intervals of 1, 2, and 3 years.

To evaluate the potential clinical utility of a prognostic signature, a nomogram was constructed based on the clinical characteristics (age, gender, stage, T, N and M) of the patient (Fig. 7A). The nomogram may aid in determining a patient’s risk with greater precision, which can ultimately inform and improve future treatment decisions. The calibration curves showed good consistency between the actual and predicted survival rates (Fig. 7B). To further assess the accuracy of the nomogram, prognostic ROC analysis was carried out, which demonstrated superior prognostic performance as compared to other clinical shapes and risk scores. The AUC values for 1-, 2-, and 3-year survival were 0.812, 0.813, and 0.825, respectively, as shown in Fig. 7C–E. These results indicate that the predictive signature has great potential as a biomarker for predicting the prognosis of CRC.

### Comparison of our gene signature with other COAD prognostic models

In order to assess the superiority of our gene signature over other COAD prognostic models, we conducted a comparison of 10 models across the entire TCGA cohort^24–33^. The 1-year AUC values for all 10 prognostic models were observed, and our findings indicated that our model incorporating the 5 gene signatures had significantly superior predictive power compared to the other 10 prognostic models (Supplementary Fig. 4).

### Functions by KEGG and GO

**Figure 8:**
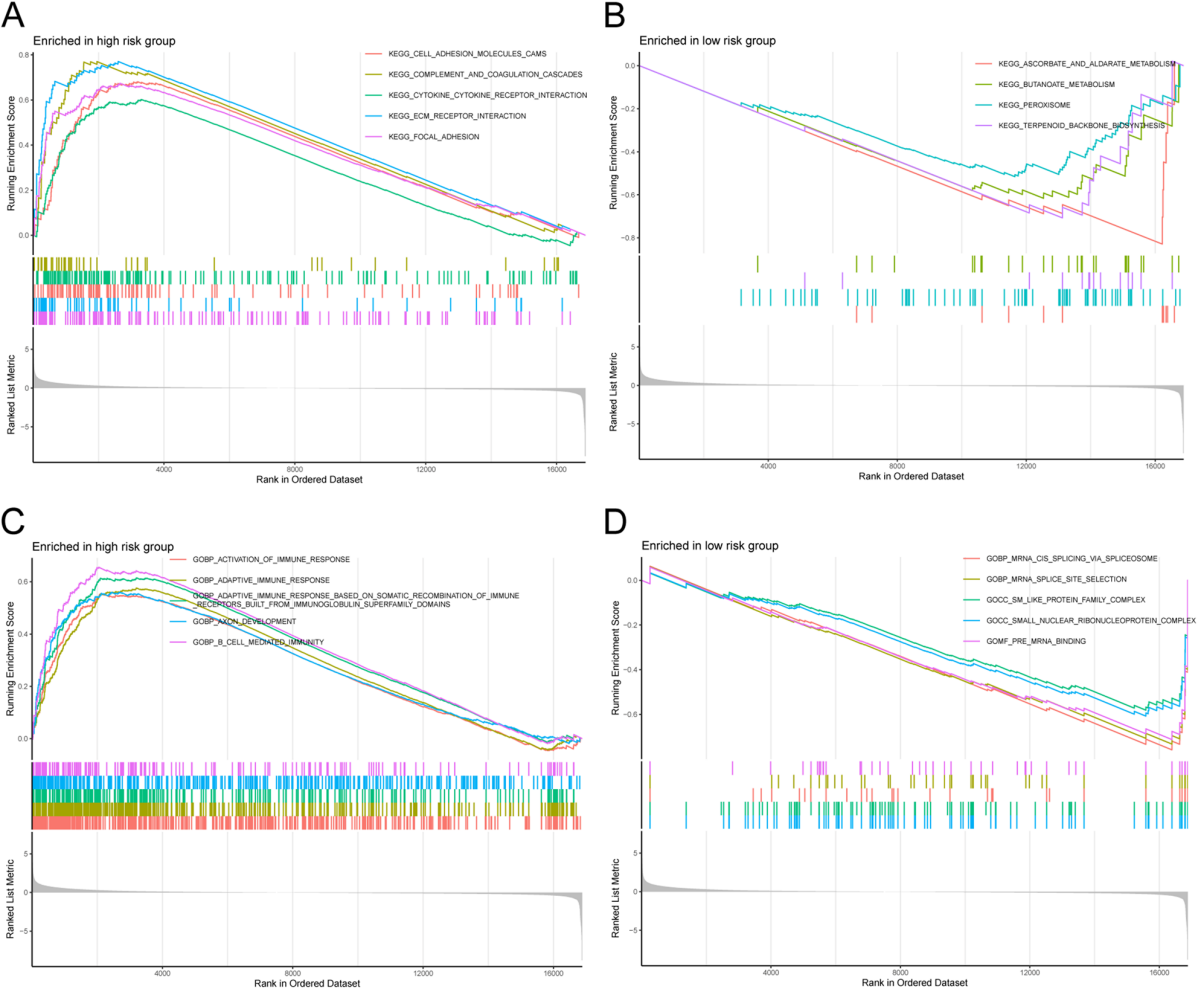
Gene set enrichment analysis. (**A**–**B**) The differentially pathways were significantly enriched in the risk groups by Kyoto Encyclopedia of Genes and Genomes (KEGG). (**C**–**D**) The differentially pathways were significantly enriched in the risk groups by Gene Ontology (GO).

The results of the KEGG enrichment analysis showed that the high-risk group expressed traits mainly related to the regulation of cell adhesion, such as extracellular matrix (ECM) receptor interaction and focal adhesion (Fig. 8A). In the low-risk group, a number of metabolic pathways were associated, including aldarate, ascorbate, and butanoate metabolism (Fig. 8B). The KEGG enrichment results have been presented in Supplementary Table 1.

In the high-risk group, GO enrichment analysis revealed that many immune-related pathways were enriched, including adaptive immune responses and B-cell-mediated immunity (Fig. 8C). However, the low-risk group was primarily associated with mRNA splicing traits (Fig. 8D). The GO enrichment results have been presented in Supplementary Table 2.

### Differences in immune infiltration between the two risk groups

**Figure 9:**
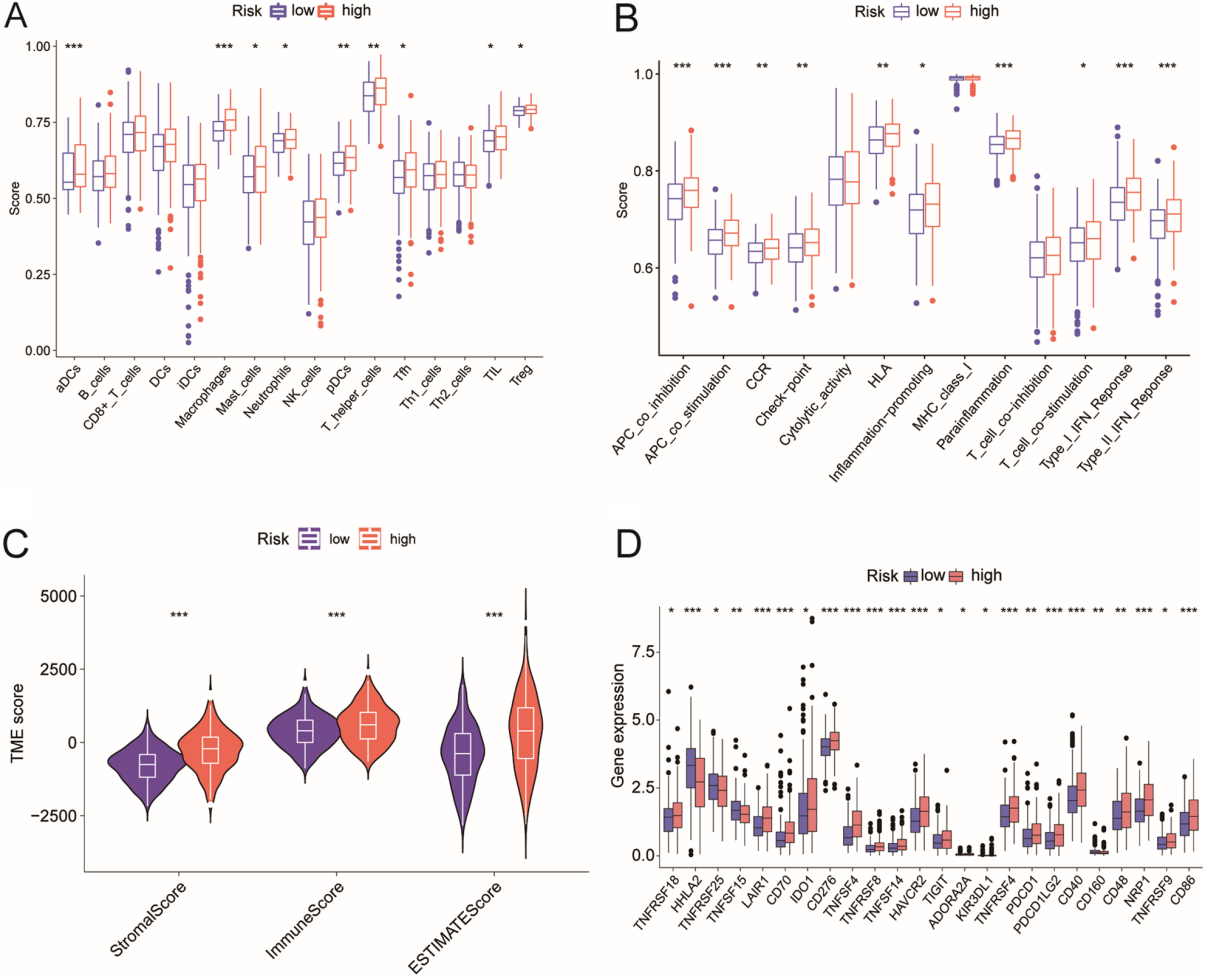
Immune infiltration differences between risk groups. (**A**–**B**) ssGSEA scores of immune cells and immune functions in the risk groups. (**C**) Comparison of StromalScore, ImmuneScore, and ESTIMATEScore between high- and low-risk groups. (**D**) The difference in immune checkpoint gene expression between the risk groups. *p < 0.05, **p < 0.01, ***p < 0.001, ****p < 0.0001.

To investigated the immune infiltration levels of different risk groups, we compiled a list of immune infiltration scores for each sample in the TCGA dataset, which is presented in Supplementary Table 3. According to the ssGSEA results, the high-risk group showed more immune infiltration and activated immune function (Fig. 9A–B). Compared to the low-risk group, the immune, stromal, and ESTIMATE scores of the high-risk group were significantly higher (Fig. 9C). In addition, immune checkpoint genes in the high-risk group were significantly upregulated (Fig. 9D). (*p < 0.05, **p < 0.01, ***p < 0.001, ****p < 0.0001).

### qRT-PCR assay in colorectal cell models

**Figure 10:**
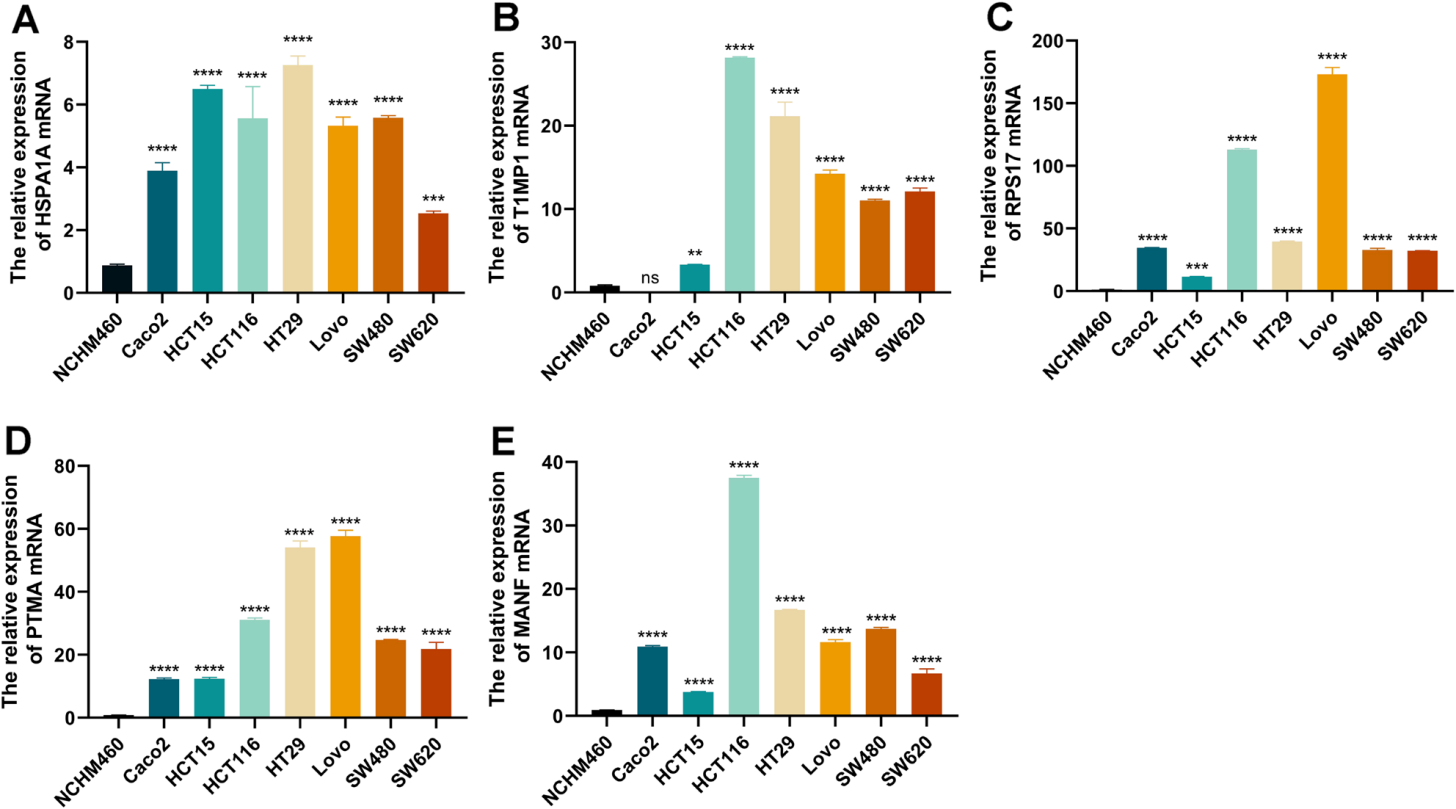
qRT-PCR analysis of predicted gene models for mRNA levels in CRC cells. (**A**–**E**) mRNA levels of HSPA1A, TIMP1, RPS17, PTMA, MANF in normal colonic epithelial cells NCHM460 versus CRC Caco2, HCT15, HCT116, HT29, Lovo, SW480, SW620. *p < 0.05, **p < 0.01, ***p < 0.001, ****p < 0.0001.

As shown in Fig. 6A–E, High expression of HSPA1A, TIMP1, and RPS17 were positively associated with poor prognosis in CRC, while PTMA and MANF’s were not significantly associated with prognosis. As shown in Fig. 10A–E, the mRNA expression levels of HSPA1A, TIMP1, RPS17, PTMA, and MANF in CRC cells were significantly elevated compared with normal intestinal epithelial cells. Among them, HSPA1A, TIMP1, and RPS17 were consistent with the clinical prognostic model we constructed, and PTMA and MANF could not be used as prognostic signature genes, which could be related to our cell specificity or insufficient sample size (*p < 0.05, **p < 0.01, ***p < 0.001, ****p < 0.0001).

## Discussion

With continuous in-depth research on ferroptosis, extensive experimental research data have shown that ferroptosis plays a vital role in tumor development. For instance, the inhibition of STAT3-ferroptosis inhibits tumor growth and alleviates chemotherapy resistance in gastric cancer^34^ Moreover, studies have suggested that cisplatin was an inducer for both ferroptosis and apoptosis in lung cancer cells^35^. However, few studies have examined ferroptosis in patients with CRC. The possible regulatory genes and markers involved in ferroptosis have not yet been fully elucidated. Therefore, a comprehensive analysis of FDRGs can contribute to the advancement of this field.

In this study, we calculated the FerrScore of different CRC-related cell types and defined low- and high-ferroptosis cell groups based on each cell type-specific median FerrScore. We also identified FDRGs in the two groups. Notably, using the TCGA database, we developed an FDRG-based risk signature to predict CRC prognosis. Five genes (HSPA1A, MANF, PTMA, RPS17, and TIMP1) were proven to have significant prognostic value. we also used qRT-PCR to test our predicted gene model and finally found that HSPA1A, TIMP1, and RPS17 were highly expressed in CRC cells and positively correlated with poor prognosis. These genes aid in distinguishing between the two risk groups. The high-risk group had a worse prognosis. Our ROC and calibration curves showed that our method accurately predicted 1-, 2-, and 3-year survival in CRC cases. To further investigate the mechanism of this method, KEGG and GO enrichment analyses were performed. As shown by KEGG analysis, the high-risk group possessed mutational traits mainly related to the regulation of cell adhesion, as demonstrated by ECM-receptor interactions and focal adhesions. Several studies have demonstrated that ECM-receptor interactions contribute to invasion and metastasis in CRC^36–38^. Our GO analysis showed that the high-risk group exhibited mutational traits mainly related to immune responses and B cell-mediated immunity. Moreover, GSVA showed that epithelial cells, monocytes, and endothelial cells were positively correlated with related pathways involved in NOTCH signaling, PI3K-AKT-MTOR signaling, and WNT-BETA-CATENIN signaling. Evidence suggests that these pathways play essential roles in the development of cancer^39–41^. Therefore, we speculated that ferroptosis may have contributed to the occurrence and development of CRC through these signaling pathways.

Ferroptosis has been linked to tumor immune infiltration. Wang et al. found that CD8^+^ T cells and fatty acids orchestrate tumor ferroptosis and immunity via ACSL4^42^. However, ferroptosis and tumor immune infiltration in CRC have not been extensively studied. We conducted ssGSEA to investigate the immune statuses of the different groups after enriching many immune-related functions in our GO study. Our results indicated that the high-risk group had increased immune cell infiltration and immune function activation. Additionally, the high-risk group had high stromal scores, immune scores, and estimated cores. Li et al.^43^ demonstrated that high immune and stromal scores are associated with poor prognosis, which is consistent with our results. Studies have shown that immune checkpoint genes are strongly associated with immunotherapy outcomes^44^. Our study revealed a general increase in the expression of immune checkpoint genes in high-risk populations. Moreover, high expression levels of these genes in high-risk patients can cause immune failure, leading to the upregulation of inhibitory checkpoint genes. Consequently, high-risk populations exhibit elevated levels of inhibitory checkpoint genes, which hold potential value as targets for immunotherapy.

The majority of genes in this signature contributed positively to the risk score, indicating that they are oncogenes. Tissue inhibitor of matrix metalloproteinase 1 (TIMP1) plays a vital role in carcinogenesis. Shou et al. have shown that TIMP1 overexpression is associated with poor prognosis in renal cell carcinoma^45^. In colon cancer, TIMP1 induces cell proliferation and invasion through the FAK/Akt signaling pathway^46^. The human ribosomal protein RPS17 plays an important role in several diseases. The mutations in the ribosomal protein S17 (RPS17) gene are closely associated with hereditary bone marrow failure syndrome^47^, while it has also been shown that RPS17 may affect CRC prognosis by controlling amino acid metabolism^48^, which is consistent with our study. The heat shock protein family A member 1A (HSPA1A) belongs to the heat shock protein 70 family. Studies have shown that HSPA1A decreases survival rates in cancers and is associated with cell proliferation and tumor grade^49^. Chen et al.^50^ confirmed that LIM and SH3 protein 1 (LASP1) and HSPA1A are both upregulated in head and neck squamous cell carcinoma, and directly bind to one another. However, few studies have explored the roles of these genes in CRC, which is an aspect where our research may provide some insights. MANF has been shown to improve colonic injury and negatively regulate macrophage polarization in colitis, indicating its potential as a therapeutic target for colonic inflammation^51^. Additionally, PTMA has been found to promote the malignant phenotype and participate in the progression of CRC, suggesting its involvement in cancer development^52^.The two studies that have shed light on the role of MANF and PTMA in colorectal cancer (CRC) development. Although the expression level of both high and low MANF and PTMA did not have a direct association with prognostic outcomes in predictive models, our study identified the high expression of five genes in CRC cells. This underscores the crucial and pressing need for additional research to further validate the predictive model and uncover more genes that could potentially serve as clinically valuable prognostic markers for CRC. Understanding the functions and interactions of the various factors involved in CRC development can help identify diagnostic and prognostic biomarkers, as well as potential targets for therapeutic intervention. As such, continued research and exploration in this field is crucial for developing effective strategies for the prevention, diagnosis, and treatment of CRC.

Overall, we identified FDRGs and developed a transcriptome-based prognosis prediction method for CRC. However, owing to the inherent complexity of tumors, other omics and experimental studies are needed to further verify the roles of these biomarkers.

In conclusion: We defined a low ferroptosis and a high ferroptosis cell group based on each cell type-specific median FerrScore. We also identified FDRGs from the two groups. The prognostic signature developed from these FDRGs demonstrated a high predictive ability for CRC outcomes.

### Supplementary Information

Supplementary Figure 1.

Supplementary Figure 2.

Supplementary Figure 3.

Supplementary Figure 4.

Supplementary Table 1.

Supplementary Table 2.

Supplementary Table 3.

## Data Availability

The datasets used and/or analyzed during the current study are available from the corresponding author on reasonable request.
