# Supplementary material for: Integrated single-cell and bulk RNA sequencing analysis identifies a prognostic signature related to ferroptosis dependence in colorectal cancer
Source: Sci Rep. 2023 Aug 4;13:12653. doi: 10.1038/s41598-023-39412-y (PMC10403602; doi:10.1038/s41598-023-39412-y)
Supplement: Supplementary file 1 — Supplementary Figure 1. [file 41598_2023_39412_MOESM1_ESM.docx]

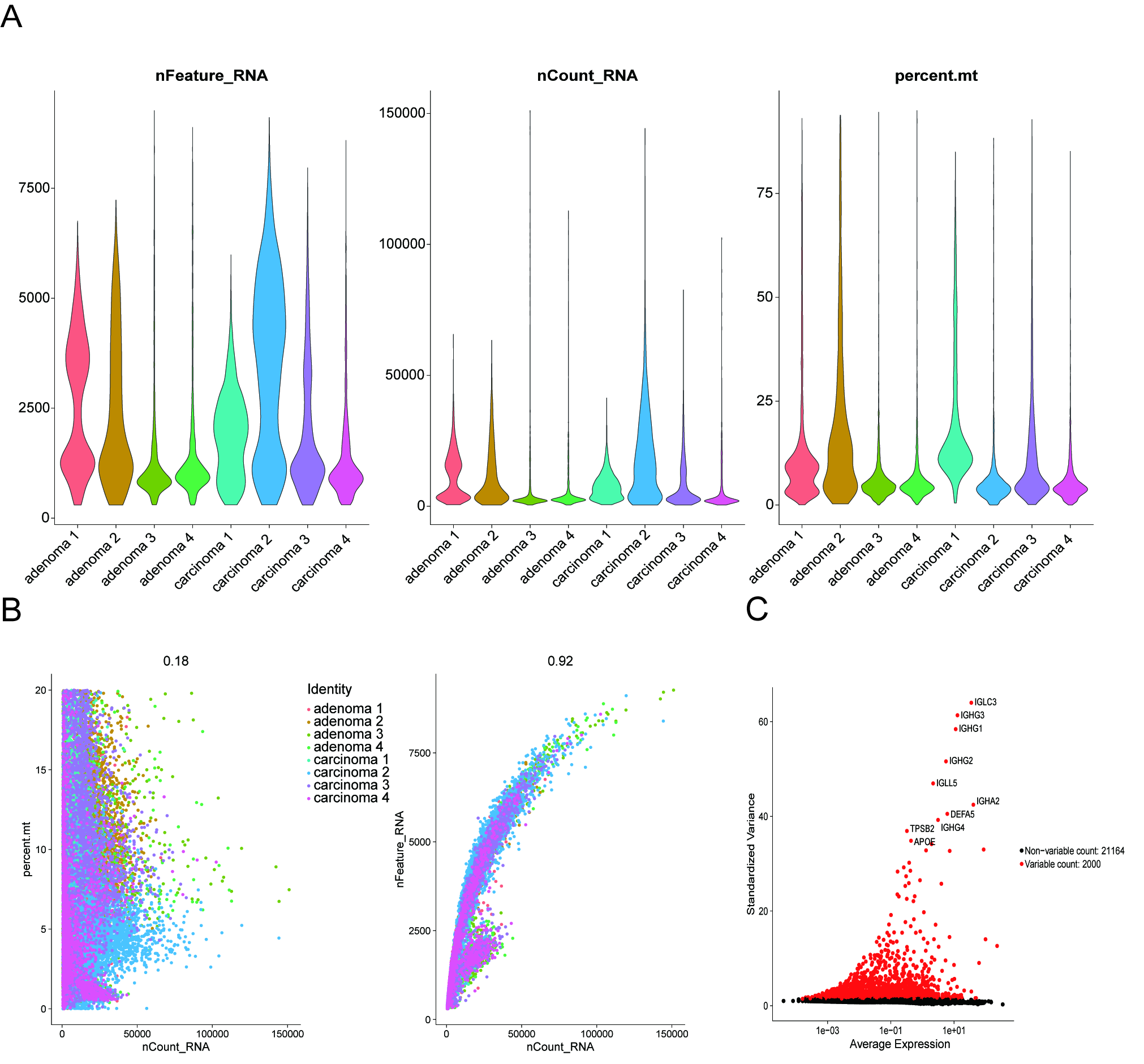


**Supplementary Figure 1.** Single-cell sequencing analysis. (**A**) Quality control filtering of each sequenced cell was performed, and a violin diagram was drawn to show its RNA characteristics (nFeature RNA), absolute UMI count (nCount RNA), and mitochondrial percentage (percente. mt). (**B**) Correlation analysis of Percente. mt, as well as nCount, nFeature, and nCount. (**C**) The top ten out of 2000 highly genes in red.
