# Supplementary material for: Integrated single-cell and bulk RNA sequencing analysis identifies a prognostic signature related to ferroptosis dependence in colorectal cancer
Source: Sci Rep. 2023 Aug 4;13:12653. doi: 10.1038/s41598-023-39412-y (PMC10403602; doi:10.1038/s41598-023-39412-y)
Supplement: Supplementary file 2 — Supplementary Figure 2. [file 41598_2023_39412_MOESM2_ESM.docx]

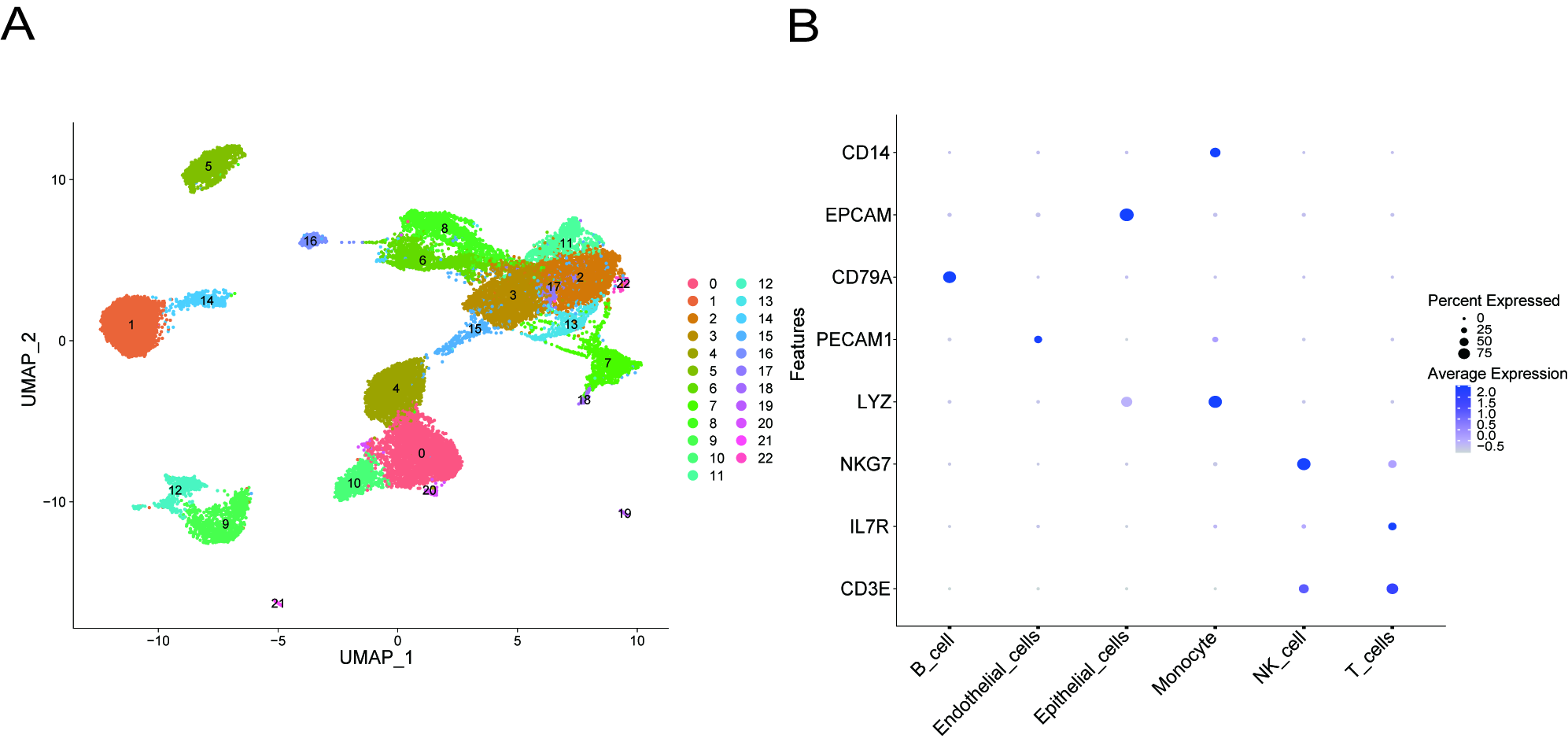


**Supplementary Figure 2.** (**A**) Identified cell types by marker gene expression and gene set variation analysis (GSVA). A UMAP was used to divide the cells into 23 clusters. (**B**) A Bubble-plot visualized marker gene expression features of each cluster in single-cell RNA sequencing profiles. Marker genes are listed on the Y-axis, and clusters on the X-axis. Dot size reflects the percentage of expression of each gene in each cluster. Dot color indicates expression level.
