# Supplementary material for: Integrated single-cell and bulk RNA sequencing analysis identifies a prognostic signature related to ferroptosis dependence in colorectal cancer
Source: Sci Rep. 2023 Aug 4;13:12653. doi: 10.1038/s41598-023-39412-y (PMC10403602; doi:10.1038/s41598-023-39412-y)
Supplement: Supplementary file 3 — Supplementary Figure 3. [file 41598_2023_39412_MOESM3_ESM.docx]

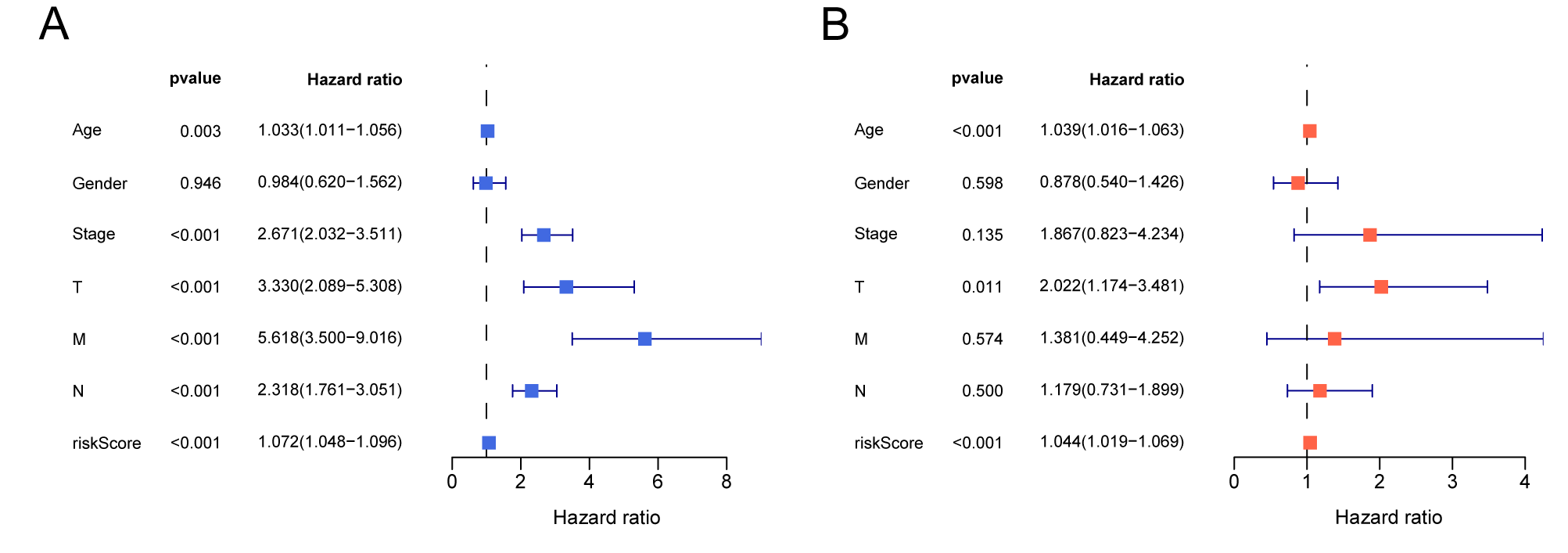


**Supplementary Figure 3.** (**A-B**) Univariate and multivariate regression analysis of the riskscore in combination with age, TNM, stage and Gender in the TCGA cohort.
