# Supplementary material for: Integrated single-cell and bulk RNA sequencing analysis identifies a prognostic signature related to ferroptosis dependence in colorectal cancer
Source: Sci Rep. 2023 Aug 4;13:12653. doi: 10.1038/s41598-023-39412-y (PMC10403602; doi:10.1038/s41598-023-39412-y)
Supplement: Supplementary file 4 — Supplementary Figure 4. [file 41598_2023_39412_MOESM4_ESM.docx]

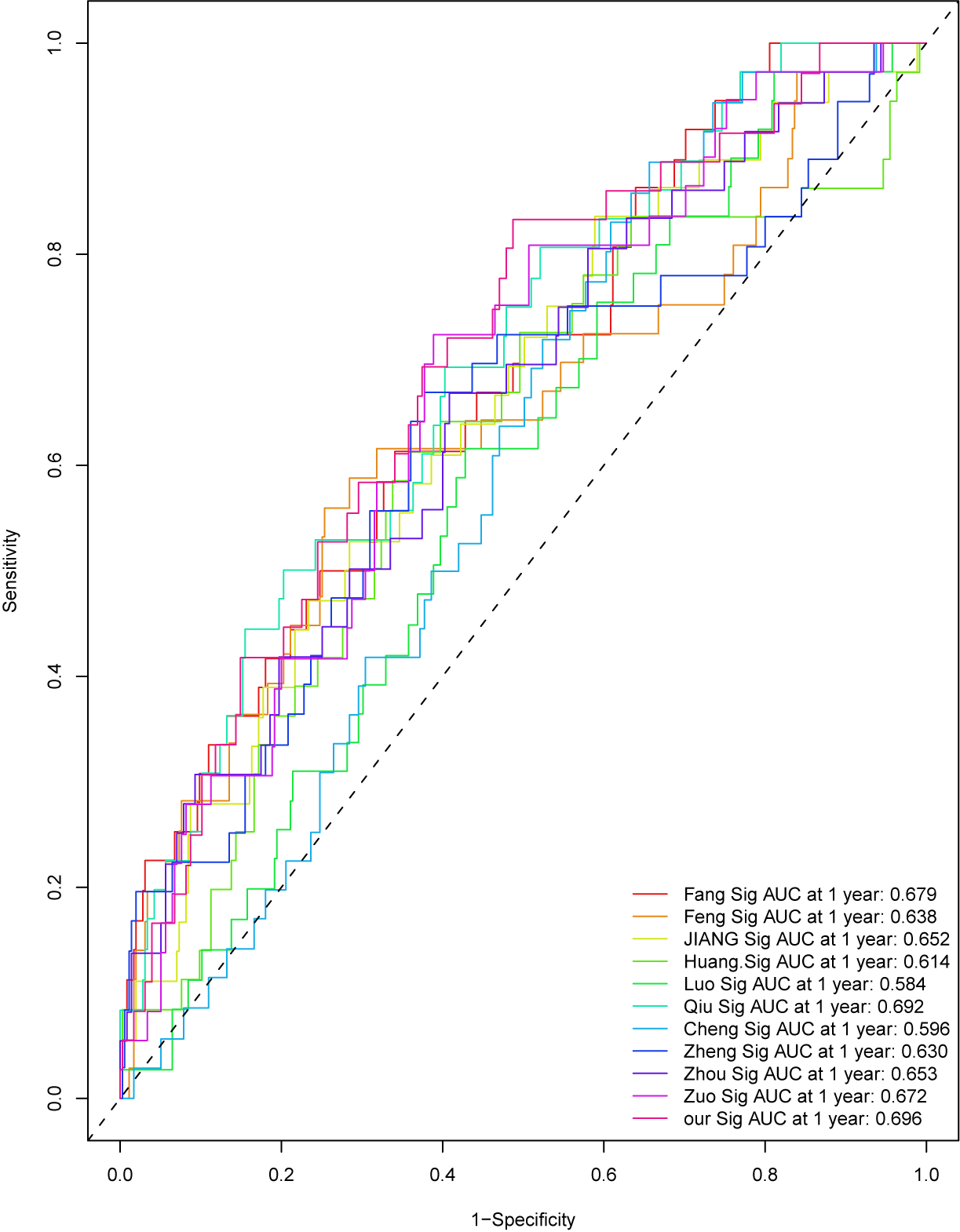


**Supplementary Figure 4.** The ROC analysis at 1 year of overall survival for our signature and Other COAD Prognostic Models in the entire TCGA cohort.
